# A Qualitative Exploration of the Determinants of Exclusive Breastfeeding (EBF) Practices in Wajir County, Kenya

**DOI:** 10.1186/s13006-020-00284-x

**Published:** 2020-05-18

**Authors:** Mahat Jimale Mohamed, Sophie Ochola, Victor O. Owino

**Affiliations:** 1 Department of Food, Nutrition and Dietetics, Kenyatta University, Nairobi, Kenya; 2 International Atomic Energy Agency, Vienna, Austria

**Keywords:** Exclusive breastfeeding, Primiparous mothers, Multiparous mothers, Determinants, Factors influencing exclusive breastfeeding, Kenya

## Abstract

**Background:**

The World Health Organization recommends exclusive breastfeeding for the first six months of life. A qualitative study was conducted to assess the factors that influence the practice of exclusive breastfeeding amongst mothers attending Wajir County Hospital, Kenya.

**Method:**

This study was part of a cross-sectional study to compare the exclusive breastfeeding rates amongst primiparous and multiparous mothers with infants under 6 months old attending Wajir County Hospital. Focus group discussions and key informant interviews were conducted to collect information on exclusive breastfeeding and related factors. Four focus group discussions were conducted with mothers who exclusively breastfed and the same number with mothers who did not exclusively breastfeed their babies. Key informant interviews were conducted with nine healthcare providers. The data were transcribed, and a content analysis identified common themes and inferences.

**Results:**

The exclusive breastfeeding rate among the mothers in the larger study was 45.5%. There was no disparity between the practice of exclusive breastfeeding between primiparous and multiparous mothers. Despite the high knowledge and positive attitudes towards exclusive breastfeeding of most mothers, the practice of exclusive breastfeeding was unsatisfactory. The major hindrances identified were cultural barriers propagated by mothers-in-law and traditional birth attendants; the belief that babies cannot live without water; and a few unsupportive health workers. The uptake of exclusive breastfeeding was enhanced by Islamic teaching on breastfeeding, education from a few supportive healthcare providers; support from husbands; and positive deviance among some lactating mothers who practiced exclusive breastfeeding.

**Conclusions:**

Deeply rooted cultural factors were the major hindrance to the practice of exclusive breastfeeding. Most of the mothers did not practice exclusive breastfeeding, despite the majority being knowledgeable and having positive attitudes towards the practice. The influence of mother-in-law’s and traditional birth assistants were major barriers. Strengthening the Community Health Strategy through training traditional birth attendants on Infant Young Child Nutrition practices, designing mechanisms linking traditional birth assistants to existing health facilities for support, and capacity building and monitoring is critical in promoting exclusive breastfeeding. Behavior change and communication through multiple channels within the community should be utilized to maximize promotion of exclusive breastfeeding among all stakeholders.

## Background

Exclusive breastfeeding (EBF) for the first six months of life and continued breastfeeding with safe, appropriate and adequate feeding is recommended as a global health policy [1]. Universal coverage of exclusive breastfeeding is estimated to prevent around 13% of all deaths among children under five years of age in low and middle-income countries [2]. Sub-optimal breastfeeding practices contribute to 11.6% of mortality in children under 5 years of age [3]. According to the 2016 Lancet series on breastfeeding, “breast is best” for lifelong health and if optimally practised has the potential to reduce the mortality of 823,000 infant deaths annually, and the greatest potential of all preventive interventions to reduce the annual infant mortality rate [4–6]. Breastfeeding provides sustainable long-term health and economic benefits to the infant, mother and the society. Failure to breastfeed is associated with lower intelligence and economic losses valued at 49% of world gross national income [5].

Globally only 38% of infants aged between 0 to 6 months are exclusively breastfed - a rate far lower than the global World Health Assembly 2025 EBF target of 50% [4]. In Kenya, the Demographic Health Survey findings indicated that the EBF rate had improved significantly from 32% in 2008 to 61% in 2014 [7]. The Ministry of Health has recently intensified nationwide breastfeeding protection and promotion interventions through strengthening the coordination and programming of Infant and Young Child Nutrition interventions. These interventions include up-scaling of the Baby Friendly Hospital Initiative (BFHI), formation of Mother to Mother Support Groups, strengthening community support mechanisms and developing a comprehensive Communication Strategy on Infant and Young Child Feeding and the Baby Friendly Community Initiative. The BFHI is a widely promoted WHO/UNICEF international programme to increase exclusive breastfeeding rates and extend breastfeeding duration. To become baby-friendly, it is mandatory for hospitals and maternity centres to practice the ten steps to successful breastfeeding. The 10thStep involves the promotion of appropriate breastfeeding practices at the community level. The BFHI, which is facility based, is limited in achieving the 10th Step as the focus is on the formation of Community Support Groups [8, 9]. Consequently, the BFCI was developed to expand the BFHI’s 10^th^ Step, focusing on support for breastfeeding mothers after they leave hospital. This initiative uses many channels to protect, promote and support breastfeeding within the community, targeting all stakeholders [9].

Despite improvement, there is wide regional disparity in EBF rates in Kenya. The EBF rate in Wajir County is 45.5% [10], whereas in Nyando district Kisumu County one-out of three (33%) infants less than 6 months old are exclusively breastfed [11]. In Kibera, an informal settlement in Nairobi, the rate of EBF was 25.6% [12].

To promote EBF it is crucial to understand the factors related to the practice. Studies in Kenya have identified various factors as potential determinants of exclusive breastfeeding. The aim of this study was to investigate the factors that influence exclusive breastfeeding among infants under 6 months in Wajir County in Kenya.

## Methods

### Study design

This study was the qualitative component of a larger cross-sectional study whose aim was to compare exclusive breastfeeding rates among primaparous and multiparous mothers [10]. Here the aim was to establish the factors related to exclusive breastfeeding practices. The qualitative study design was selected to allow in-depth understanding of the participants’ feelings, values and perceptions that underlie and influence their behaviour towards the feeding of infants. Focus Group Discussions (FGDs) and Key Informant Interviews (KIIs) were used to collect data on the study participants’ perceptions on factors related to the practice of EBF. The study received ethical approval from Kenyatta University Ethical Review Committee.

### Study setting and study participants

The study was carried out at the Maternal and Child Health (MCH) Clinic, Wajir County Hospital, Kenya. Wajir County has a rural-urban population whose main livelihood is pastoralism. The area is predominantly inhabited (over 90%) by the ethnic Somali community. The Somali community are predominantly Muslims and share common cultural practices that impact on breast and infant feeding [13, 14]. These practices include giving pre-lacteals, early initiation of complementary feeding practices, delayed initiation of breastfeeding, negative attitudes towards the consumption of colostrum and the perception that mothers’ breastmilk is inadequate for the baby. Wajir is ranked the 45th poorest County out of 47 Counties in Kenya. Despite the County recording one of the highest birth rates at 7.8 births per woman compared to the average national rate of 3.9 births per woman [7], the County has the lowest rate of health facility deliveries (18%) and is rated as one of the Counties with the highest maternal mortality rates in Kenya with 1687 maternal deaths per 100,000 live births [15].

The study participants were mothers of infants under 6 months old attending the Maternal and Child Health Clinic at Wajir County Hospital, during the study period. The mothers enrolled in the focus group discussions (FGDs) had participated in the larger cross-sectional study [10]. The exclusion criteria were the same as for the larger study, that is, mothers with medical conditions or on medications which contraindicated breastfeeding, and those with infants diagnosed with serious congenital malformations where breastfeeding was not feasible or contra-indicated. Mothers were recruited into the study upon giving informed consent.

### Sample size and sampling procedure

**Fig. 1:**
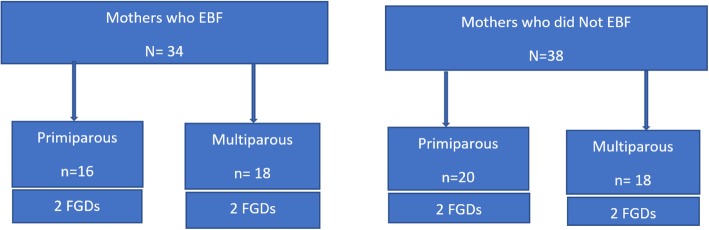
Focus Group Discussions sample size

Four FGDs were conducted with the mothers who exclusively breastfed (*N* = 34), and four with those who did not exclusively breastfeed (*N* = 38), making a total of 72 mothers. Each category of mothers was further sub-categorized into primiparous and multiparous groups. See Fig. 1. The participants were randomly selected using a Table of Random Numbers.

A total of nine Key Informant Interviews (KIIs) were conducted with nine health care providers at the Wajir County Hospital. The health care providers included nurses, clinical officers and nutritionists. The Maternal Child Health nurse in-charge, county clinical officer and chief nutrition officer were among those interviewed. The selection criterion for the health providers was limited to those who offered health services to children at Wajir County Hospital. The researcher explained the objectives of the research, interview protocols and informed consent.

### Data collection procedure

FGD guides were used to collect information on the factors that influenced the practice of EBF in the study region. Two FGD guides for mothers who exclusively breastfed, and two for mothers who did not exclusively breastfeed (one for multiparous and another primiparous mothers respectively) were used to collect the data. The FGD guides were developed in English, translated to Somali and then back into English to ensure accuracy of the translation. The guides were pre-tested among six primiparous women who practiced EBF and seven multiparous women who did not practice EBF.

An appropriate time and venue for the focus group discussions were agreed upon by the researcher and the participants. The FGDs were facilitated by the researcher assisted by two research assistants who recorded the deliberations and acted as an observer, respectively. The FGDs were conducted in a private, quiet room at the MCH clinic, Wajir County Hospital. The researcher first explained the objectives of the FGDs and encouraged everybody’s participation in the discussion. The discussions focused on maternal knowledge, attitudes and practices of EBF, as well as soliciting information on factors related to the practice of EBF. The FGDs lasted between 60 and 90 min each and the discussions were tape recorded.

A key informant interview guide was developed in English and not translated into Somali because all the health care providers were proficient in English. The KII guides were pretested for clarity and validity among health service providers at Wajir County Hospital not recruited for the larger study. All the KIIs were conducted by the researcher and attended by a research assistant who tape-recorded the deliberations. The interviews focused on the mothers’ knowledge, attitudes and practices of EBF; factors related to the practice of EBF; and the challenges mothers face in the practice of EBF. Data saturation was achieved after a total of four focus groups were held; two with the primiparous mothers and two with the multiparous mothers.

### Data analyses

A content analysis of the data from the focus group discussions and key informant interviews was undertaken. The content analysis involved a detailed exploration for common themes and assigning labels to variable categories. The categories or themes were identified or predetermined in advance. The themes were in line with the objectives of the research, that is, to identify enablers and hindrances to the practice of exclusive breastfeeding. The coding consisted of searching for common themes which were established as categories under which information was later inserted. The themes were clustered in a patterned order to identify variables to enable general concepts and isolated repetitions to be identified. Care was taken to ensure that a categorization agreed with the context it was taken from. Inferences were made from the data under the following sub-themes: factors enabling EBF (maternal knowledge and positive attitudes towards EBF, spousal support, and encouragement from heath care providers); and factors hindering the practice of EBF (negative attitudes towards EBF, and socio-cultural beliefs and practices). Conclusions were drawn from the findings.

## Results

### Maternal socio-demographic characteristics

A total of 72 mothers took part in the FGDs (those who EBF = 34; those who did not EBF = 38). The mean age of the mothers was 25.8 years (range 17–39 years). The majority of the primiparous mothers who exclusively breastfed were aged between 18 and 25 years, whereas the mean age for multiparous mothers who exclusively breastfed was 31 years. Almost all the mothers from both groups were married. Amongst the mothers who exclusively breastfed, most primiparous mothers had attained primary education while most of multiparous mothers had no formal education. The mothers who had not exclusively breastfed (both primiparous and multiparous) had received formal education (Table 1). Of the nine healthcare providers interviewed, five were nurses, two were clinical officers and two were nutritionists (Table 2). The majority (44.5%) of the healthcare providers had diploma level training in their specialty, with over half aged between 31 and 40 years (Table 3).

**Table 1:** Maternal socio-demographic characteristics

Aspects	Mothers who did EBF		Mothers who did not EBF	
	N = 34		N = 38	
	P*(***n*** = 16)	M* (***n*** = 18)	P*(***n*** = 20)	M* (n = 18)
**Maternal age**				
< 18 Years	2	2	2	0
> 18–25 years	8	2	10	2
26–30 years	4	6	6	4
31 years and above	2	8	2	12
**Marital status**				
Married	14	14	18	7
Single	0	0	0	0
Divorced	2	2	0	1
Separated	0	0	0	0
Widow	0	2	2	1
**Education**				
No formal education	8	12	4	8
Adult education	0	0	0	0
Primary education	2	4	8	0
Secondary school	4	2	4	6
Certificate level	0	0	2	2
Diploma level	2	0	2	2
Degree level	0	0	0	1

P*: Primiparous mothers; M*: Multiparous mothers

**Table 2:** Characteristics healthcare service providers

Aspects	*N* = 5	
	Number (n)	%
**Age**		
20–30 yearsz	3	33.3
31–40 years	5	55.6
41 & above	1	11.1
**No. of years in service:**		
1–2 years	1	11.2
3–5 years	4	44.4
6 years & above	4	44.4
**Level of Education:**		
Certificate	2	22.2
Diploma	4	44.5
Degree & above	3	33.3
**Specialty**		
Nursing	5	55.6
Nutrition	2	22.2
Clinical officer	2	22.2

**Table 3:** Summary of the Focus Group Discussion (FGDs) findings on the determinants of exclusive breastfeeding

Enablers of EBF	Barriers to EBF
• High knowledge on EBF • Maternal positive attitude towards exclusive breastfeeding. • History of maternal MCH attendance and hospital delivery • Breastfeeding counselling provided at the health facilities • Spouse’s (husband0 support) • Religious reasons - Breastfeeding is a mandatory spiritual calling ordained in the Holy Quran. • Positive variance – influence of other experienced • Self-belief	• Negative socio-cultural factors e.g. TBA’s and • grandparents/mother-in-law’s influence • Negative maternal attitude towards exclusive breastfeeding • Maternal home deliveries • Caesarean section as mode of delivery • Non –supportive healthcare providers

### Summary of focus group discussions (FGDs) findings

#### Factors enabling the practice of EBF

The mothers had high knowledge of breastfeeding. Most had a good understanding of the concept of exclusive breastfeeding and indicated that it meant giving the baby only breast milk without even water for six months. Almost all the mothers had a good understanding of the benefits of EBF for the child and the mother. One primiparous mother who exclusively breastfed her infant said, “*Breastmilk has all the nutrients an infant requires, it is not contaminated and will make the child grow well as required”.* The same sentiment was echoed by almost all the mothers who practiced, and those who did not practice EBF. One multiparous mother reported, “*Breastmilk does not cause diarrhea and vomiting as animal milk sometimes does. Furthermore, breastfeeding prevents pregnancy especially when actively breastfeeding”.* The high level of maternal knowledge on EBF reported by most mothers indicated they had adequate information to choose whether to practice EBF and this was an important influence on their feeding decision (Table 3).

Most mothers held positive attitudes towards EBF and acknowledged that breast milk is irreplaceable and should be fed to babies as a priority. One mother pointed out, *“Breastmilk is superior to all other feeds and is a gift from God that all mothers should not forget to utilize”.* Others acknowledged that breastfeeding is prescribed in the Holy Quran and failure to breastfeed had spiritual consequences. Since almost all the study participants were Muslims, this belief encouraged mothers to breastfeed, although it may not necessarily have influenced all the mothers to practice EBF. Nevertheless, this unanimous view was indicated through comments such as “*I believe a mother can exclusively breastfeed her child for six months without any supplement including water. I have done it for the third time now”.* These positive attitudes encouraged others to practice EBF. Health care providers also positively influenced the practice of EBF amongst the mothers who regularly sought services at the Ante Natal Care and Maternal Health Care on a regular basis, and those who delivered their babies at a health facility. Some husbands were very supportive and encouraged their wives to breastfeed exclusively. Furthermore, children of the mothers who practiced exclusive breastfeeding grew healthily and had a much lower morbidity rate which encouraged others to breastfeed their children.

#### Factors hindering the practice of EBF

Socio-cultural factors were reported to hinder the practice of EBF. Such factors are deeply rooted in the Somali community and play a major role in encouraging mothers not to practice exclusive breastfeeding. For instance, when asked whether she believed a baby can survive on breast milk alone, without even water, for the first six months, one multiparous mother stated, “*Because of the hot harsh weather conditions in Wajir County, a child needs some water to quench thirst. Some mothers do not produce enough breast milk and this trend is prevalent in some families.”* Other mothers held similar views. One primiparous mother stated, “*On delivery the child needs some water to dilute the colostrum and clear the system.”* Similarly, a mother of four indicated that a “*Majority of the mothers believe that if a woman breastfeeds while pregnant, her milk will be toxic and can make the baby ill and can even kill.*”

Other barriers to EBF included a mother becoming pregnant while breastfeeding and having to wean. The mothers indicated that culturally, breastfeeding during pregnancy is not acceptable and ceases when a woman conceives another child. Many cultural barriers were propagated by TBAs, grandmothers and community members. For example, the belief that baby’s first feed should be animal milk and that babies should be given water to quench thirst and to dilute colostrum which they viewed as harmful to the baby because it is too strong. Consequently, the mothers experienced immense pressure from grandmothers and TBAs to give pre-lacteals and animal milk to infants aged under six months. For example, one mother who dropped out school at Standard eight said, “*I had immense pressure from my mum-in-law and my husband to introduce water and cow’s milk as early as the first week to my first child. I resisted this having understood the importance of EBF. Luckily my child grew well with no hospital visits compared to my neighbour’s child who was given cow’s milk and water early. My husband and mother-in-law are now convinced that EBF has health benefits for the baby and I… did not have a tussle of war on how to feed my subsequent children*”.

Most of the mothers who delivered at home assisted by TBAs reported being pressured to administer pre-lacteal feeds such as cow’s milk, water, honey, cow/goats’ milk and sugar/glucose to their babies as early as the first day after delivery [16]. Additionally, grandmothers and mothers-in-law greatly influenced the introduction of pre-lacteals. In a region where majority of mothers opt for home delivery assisted by TBAs, cultural factors override the knowledge received from health professionals on EBF. Most mothers preferred to deliver their babies at home for reasons such as unfriendly health care providers and inaccessible health facilities. One primiparous mother who delivered her a baby in Wajir County hospital and routinely took her child to the Maternal Health Clinic, reported several unpleasant experiences with healthcare providers: “*The nurses and clinicians are not friendly and are always in a hurry. If you ask them questions, they get irritated and put you off. If only I had on option of having my child immunized elsewhere with free government vaccines, I would not go to the health facility at all.”*

The mothers who preferred home delivery confirmed their familiarity with the TBAs and their understanding of their circumstances and culture. One mother said, *“I delivered all my three children at home under the care of an experienced TBA, as advised by my grandmother. Since then, the experience has been good, my daughters and those of my sisters will follow the same.”*

Mothers who delivered through cesarean section at health facilities reported initiating pre-lacteals early. One multiparous mother who delivered her two sons in hospital by cesarean section said, *“In the hospital, after my delivery… the nurses started feeding the child with water and glucose and/or powder milk, making the child refuse the breastmilk/breast attachment, and this reduced my milk production.”* These sentiments were overwhelmingly supported by majority of the mothers who delivered through cesarean section. Nonetheless, these mothers represented the small percentage (less than 2%) who delivered through cesarean section and were not a major factor influencing the practice of EBF. Nonetheless, it was reported that the majority of those who practice EBF delivered their babies at the health facility where they were encouraged by the health workers.

### Findings from the key informant interviews on the determinants of the practice of EBF

A summary of the findings on determinants/factors influencing EBF practices as well as suggestions for improving EBF is presented in Table 4. The findings of the key informant interviews on the determinants of EBF practice among the mothers in Wajir County were consistent with those reported by mothers who took part in the focus group discussions.

**Table 4:** Summary of the Key Informant Interviews on the determinants of exclusive breastfeeding

Enablers of EBF	Barriers to EBF	Healthcare providers suggestions on improving EBF practices
• High knowledge on EBF • Maternal positive attitude towards exclusive breastfeeding. • Breastfeeding counselling provided at the health facilities and • Supportive husband/spouse • Religious reasons	▪ Negative influence of TBAs ▪ Influence by family/friends and especially grandparents and in-laws ▪ Lack of adequate support from healthcare providers ▪ Negative cultural factors ▪ Lack of support from the fathers/husbands ▪ Peer pressure ▪ Health care providers constraints and shortages	▪ Building skills and capacity of the TBAs and linking them to the health facilities. ▪ Improving capacity of healthcare providers & institutions. ▪ Increasing the number of nurses in the health facilities. ▪ Enforcing the BFHI’s in both the public and private hospitals. ▪ Training and empowering breastfeeding support groups ▪ Training and empowering breastfeeding volunteers and linking them to the health facilities

### Maternal knowledge and attitudes on the benefits of exclusive breastfeeding and its influence on the practice of EBF, reported by health care providers

The health care providers indicated that maternal knowledge on EBF was very high, although this knowledge did not always translate into practice. Similarly, the mothers’ attitude towards EBF was positive, but the practice was hindered by cultural factors. *“The concept of exclusive breastfeeding is quite familiar to all mothers and breastfeeding is encouraged, even mentioned in the Holy Quran. The challenge is implementation, and this has to do with family members, TBAs and relatives,”* said one nurse. “*The mothers, especially the first-time mothers who are determined to EBF their children, are influenced by their grandmothers and friends at home to do otherwise,”* said a clinician who had worked at the health facility for over 7 years. The health providers also acknowledged that maternal knowledge and positive attitudes encouraged some mothers to practice EBF despite cultural factors against the practice.

### Healthcare providers’ understanding of the influence of maternal sources of breastfeeding information on EBF practices

The health workers reported several sources of breastfeeding information, ranging from immediate family members such as mothers and grandmothers, TBAs, healthcare providers at Maternal Child Health, hospitals and clinics through to community health workers and the media. According to health practitioners, the information gathered from TBAs and parents was not always scientifically sound and could negatively influence breastfeeding practices.

### Healthcare providers’ knowledge and attitudes on exclusive breastfeeding and their influence on EBF practice

Overall, the healthcare providers had an excellent understanding of EBF. Their attitudes towards EBF were also positive with two nurses reporting they exclusively breastfed their infants for the recommended 6 months despite being working mothers. *“I have exclusively breastfed all my three children despite undertaking my duties like any other full-time employee. I express breastmilk even while working at the clinic and I store the expressed breastmilk in the fridge. I have involved my husband on the same and he is now more informed on EBF than me. My mother-in-law tried to influence me to introduce pre-lacteals early especially my first child, but I stood my ground.”* One of the clinicians stated*, “I am fully involved and motivated in helping my wife to exclusively breastfeed our children and that has given her all the moral and psychological support to practice exclusively breastfeeding. Cases of upper respiratory infections and diarrheal diseases are uncommon among my children.’*

### Challenges experienced by mothers and their influence on the practice of EBF reported by health providers

According to the healthcare providers, breastfeeding mothers experience several challenges in their quest to practice exclusive breastfeeding. These challenges included: information/knowledge gaps especially for mothers who do not attend the Anti-Natal Clinic frequently; inaccurate information from sources such as TBAs and family members; problems with positioning and attachment of the baby to the breast; poor nutritional status among nursing mothers; and inadequate support from healthcare providers and husbands.

### The influence of health providers’ practices on exclusive breastfeeding practices

Health care providers’ practices at the health facility positively influenced mothers who sought services at the Antenatal Care and the Maternal Child Health clinics on a regular basis, and mothers who delivered their babies at the health facility. The health providers provided counseling on the benefits of breastfeeding to the baby and the mother; relevant breastfeeding information; demonstrated how to attach the baby to the breast; and how to deal with breast problems such as engorged breasts.

### Factors related to health care providers’ constraints/challenges to influence the practice of EBF

The healthcare providers reported their support in improving EBF rates was not satisfactory due to several factors. First, the number of healthcare providers was inadequate for the large number of clients. Second, the long distance to health facilities for most mothers and the minimal support offered by the hospital management for the baby friendly hospital initiative. *“The health providers are not motivated to go an extra mile even while attending to clients because of high workloads due to limited staff”*, said one nurse. The nurses also mentioned that because mother-to-mother support groups had not taken root in Wajir, there was a need to implement, monitor and link support groups to the health facility.

### Socio-cultural factors that influenced the practice of exclusive breastfeeding

The findings of the key informant interviews that socio-cultural factors hinder the practice of EBF reflected those identified by the mothers in the focus group discussions. The health providers indicated that the practice of EBF in the community was not satisfactory and identified socio-cultural factors as a major hindrance of the practice of EBF in Wajir County.

## Discussion

The objective of this qualitative study was to explore factors (both enabling and hindering) related to the practice of EBF in Wajir County, Kenya.

### Enablers of exclusive breastfeeding

#### Maternal knowledge on exclusive breastfeeding

During the focus group discussions, the mothers indicated that a high knowledge of EBF enhanced the practice for some - a finding that was corroborated by the health providers. The high level of breastfeeding knowledge among these mothers alswso reflected government interventions aimed at promoting EBF at the national, health facility and community levels. Two strategies to promote exclusive breastfeeding during the first six months of baby’s life were the Baby Friendly Hospital Initiative and the establishment of Mother-to-Mother Support groups. Similar studies have reported high maternal knowledge in Kenya [17, 18] where knowledge is considered a pre-requisite for appropriate practice. However, key messages to promote EBF should go beyond the provision of knowledge and address the barriers to breastfeeding.

#### Maternal attitudes on exclusive breastfeeding

Overall maternal attitudes towards exclusive breastfeeding were positive. Nonetheless, there were some mothers who continued to embrace negative cultural perceptions and beliefs on EBF. These mothers claimed that breastmilk is toxic and poisonous to the baby when a mother is pregnant, and they should stop breastfeeding once pregnant with another child. Others indicated that the infant needs water in addition to breastmilk, especially in hot weather conditions. The study showed that some young mothers who delivered at home assisted by TBAs followed the footsteps of their mothers’ and grandmothers’ infant feeding practices. These findings are consistent with those from Bangladesh where young mothers’ attitudes towards and infant feeding practices were influenced by older women in their communities whose attitudes/perceptions and practices were based on cultural beliefs [9]. Cultural attitudes, beliefs and norms are important factors in the WHO’s model of the determinants of infant and child feeding behavior [19], as they influence breastfeeding practices. In Lebanon, detrimental cultural beliefs surrounding infant and young child feeding practices such as perceptions of insufficient milk and “bad” colostrum were shown to influence maternal attitudes [20]. Strategies to promote the practice of EBF need to address those cultural practices that hinder the adoption of the practice.

#### Health care services provided at the health facilities

Hospital delivery and Maternal Child Health clinic visits played a key role in the practice of EBF in the community. The mothers who regularly attended Maternal Child Health and Antenatal Clinics and those who delivered at health facilities were positively influenced to exclusively breastfeed by the healthcare providers. Given the relatively low proportion of mothers who deliver at health facilities [7], empowering the community on the health benefits of delivering at a health facility should be emphasized. This emphasis would serve the dual purpose of ensuring safe deliveries while at the same time empowering women to adopt appropriate infant feeding practices. Studies have shown that women who receive encouragement and support on breastfeeding from health care providers are more likely to initiate and sustain breastfeeding [21–23].

#### Support for breastfeeding from family members

Maternal breastfeeding support was reported to have encouraged mothers to exclusively breastfeed, particularly support received from husbands and mothers. These findings were consistent with those of other studies [24–26]. Similar findings were also reported in low socio-economic Tijuana, Mexico [27]. Additionally, mothers reported that the Holy Quran’s recommendation to breastfeed their children for at least two years was a source of motivation to EBF their children. Other studies also strongly demonstrated the key role of the Holy Quran in the practice of EBF, especially amongst the Muslim community [28–30].

### Barriers to exclusive breastfeeding

Maternal socio-cultural factors negatively influenced the adoption of EBF. In Kenya, ingrained cultural beliefs among certain communities such as a long duration breastfeeding, are associated with sagging breasts and ‘evil eye” (malevolent glare) particularly if a mother breastfeeds in public [16, 31]. Such beliefs were reported to be detrimental to the child’s health and development and to negatively impact on the practice of EBF. Maternal sources of information also influenced the practice of EBF. Traditional birth attendants were identified as the main source of breastfeeding information despite propagating cultural practices such as early administration of pre-lacteals to infants. Since traditional birth attendants are largely respected by the community and conduct most of the deliveries, the Ministry of Health and Non-Government Organizations should endeavor to build the capacity of traditional birth attendants through monitoring and supporting their activities and linking them to existing health facilities. Older women, particularly grandmothers, also contributed to the low adoption of EBF in this community through prevailing upon younger mothers to practice traditional infant feeding practices. It was also reported that some husbands were unsupportive of their wives and so hindered the adoption of EBF practices. These findings confirm that the promotion of EBF should not only target mothers, but all stakeholders, including grandmothers and fathers.

Health providers to some extent also contributed to the low adoption of EBF. The mothers reported that some health providers were unfriendly and did not have patience to address the concerns of their clients. The health providers interviewed acknowledged they lacked adequate time to promote appropriate breastfeeding practices because of a high workload with large numbers of clients. The mode of delivery also played a role in exclusive breastfeeding practices with early initiation of pre-lacteals largely reported amongst mothers who underwent a cesarean section.

## Conclusion

The enablers of EBF among mothers in this study were high levels of knowledge and positive attitudes towards EBF. Supportive husbands and some health workers also encouraged the practice of EBF, even though the rate was low. Maternal socio-cultural factors surrounding breastfeeding practices propagated by traditional birth attendants and mothers-in-law contributed to the low EBF rates in the study community. Strategies to enhance the uptake of EBF should maximize existing community support such as traditional birth assistants and mothers-in-law. With the high involvement of traditional birth assistants in deliveries and early dissemination of breastfeeding information to mothers, the government and other stakeholders should investigate appropriate and feasible mechanisms to link traditional birth assistants to health facilities for support, capacity building and monitoring. Behavior change and communication through multiple channels throughout the community should be used to maximize promotion of the practice of EBF to target all stakeholders, particularly fathers and grandmothers. We recommend further research to investigate the feasibility of linking traditional birth assistants to the health facilities and its effect on the practice of EBF in the community. One strength of the study was that the participants, as members of the same ethnic community, shared cultural values and practices. Consequently, once appropriate interventions are identified and developed, they are likely to make a positive impact on the breastfeeding rates throughout the County. A key limitation of this study is that the region was purposively selected and mainly inhabited by one community, Kenyan Somalis. This factor limits generalization of the findings to the other communities in Kenya. A further limitation was that the rates of EBF were dependent upon maternal self-reporting based on a 24-h recall period, which may have introduced recall bias.

## Data Availability

The datasets during and/or analyzed during the current study available from the corresponding author on reasonable request.

## Abbreviations

BFHI: Baby Friendly Hospital Initiative
EBF: Exclusive Breastfeeding
FGD: Focus Group Discussion
KIIs: Key Informant Interview
WHO: World Health Organization
